# CIRBP is a novel oncogene in human bladder cancer inducing expression of HIF-1α

**DOI:** 10.1038/s41419-018-1109-5

**Published:** 2018-10-12

**Authors:** Mengxin Lu, Qiangqiang Ge, Gang Wang, Yongwen Luo, Xiaolong Wang, Wei Jiang, Xuefeng Liu, Chin-Lee Wu, Yu Xiao, Xinghuan Wang

**Affiliations:** 1 Department of Urology, Zhongnan Hospital of Wuhan University, Wuhan, China; 2 Department of Biological Repositories, Zhongnan Hospital of Wuhan University, Wuhan, China; 3 Human Genetics Resource Preservation Center of Hubei Province, Wuhan, China; 4 Laboratory of Precision Medicine, Zhongnan Hospital of Wuhan University, Wuhan, China; 5 Department of Urology, Ludwig-Maximilians-University Munich, Munich, Germany; 6 Medical Research Institute, Wuhan University, Wuhan, China; 7 Department of Pathology, Lombardi Comprehensive Cancer Center, Georgetown University Medical School, Washington, DC USA; 8 Department of Urology, Massachusetts General Hospital, Harvard Medical School, Boston, MA USA

## Abstract

Cold-inducible RNA binding protein (CIRBP) has been reported to be associated with distinct tumorigenesis. In this study, we investigated the role of CIRBP in human bladder cancer (BCa), indicating that CIRBP is overexpressed in BCa tissues and cell lines to promote proliferation and migration. Moreover, CIRBP could induce expression of HIF-1α via binding to the 3′-UTR of its mRNA to increase the mRNA stability in BCa cells. Furthermore, we demonstrated that PTGIS is a HIF-1α targeted gene, a major regulator in hypoxic cancer progression by activating transcription of various oncogenes. Our results also suggested that overexpression of HIF-1α may suppress the expression of PTGIS in BCa cells, by binding to HRE sequence at the promoter region of PTGIS. In addition, we found a strongly downregulation of PTGIS in BCa tissue and transcriptionally inhibited by HIF-1α in BCa cells, which could be triggered by its DNA methylation. Further result suggested that knockdown of CIRBP could promote the expression of PTGIS, meanwhile knockdown of PTGIS could partially rescue CIRBP-deficiency induced inhibition of migration and proliferation in BCa cells. Taken together, our study indicated that CIRBP could be a novel oncogene in human bladder cancer inducing transcription of HIF-1α, which could inhibit expression of methylated PTGIS.

## Introduction

Bladder cancer (BCa) is the ninth most common malignancy worldwide, with an estimated 429,000 new cases and 165,000 deaths per year in the world^1^. Recent studies have evaluated key molecular pathways of BCa, such as RAS–mitogen­activated protein kinase (MAPK) pathway, Wnt/β-catenin signaling pathway, and epithelial–mesenchymal transition (EMT)^2^. Cold-inducible RNA binding protein (CIRBP) was originally identified in the testis as a mammalian cold shock protein^3^, and is induced by cellular stresses such as UV radiation, cold and hypoxia^3–6^. CIRBP has been reported to be overexpressed in several human tumors, such as prostate, colon, breast and skin carcinomas^7–9^. Recent mechanistic studies show that CIRBP displayed the ability to bypass replicative senescence through activation of the ERK1/2 signaling pathway in primary mouse embryonic fibroblasts^7^, further evidence for CIRBP’s oncogenic function is that CIRBP promotes the development of liver cancer by increasing ROS accumulation and CD133 expression^10^. However, the involvement of CIRBP in BCa has not yet been investigated.

Recent studies describe that the stabilization of mRNAs by specific RNA binding proteins (RBPs) can affect the rate of translation in response to stress^4,11^ or extracellular signals^12^. Recently, several important studies^13,14^ have reported that Y-box binding protein 1, an RNA-binding protein, enhances HIF-1α protein expression by directly binding to and activating translation of HIF-1A messages. CIRBP is an RNA-binding protein and binds specifically to the 3′-untranslated regions (3′-UTR) of many target mRNAs^4,15,16^, and affects their post-transcription expression. It was described that CIRBP can increase in response to hypoxia by a HIF-1α-independent mechanism^6^, moreover, our results demonstrate that CIRBP can increase HIF-1α expression in BCa. In addition, Yang et al. reported that HIF-1A is one of CIRBP-targeted transcripts in the UniGene 3′-UTR data base^15^. These results indicate that CIRBP may increase mRNA stability and protein translation of HIF-1α by binding to the 3′-UTR of its mRNA transcripts.

In human cancers, overexpression of HIF-1A in tumor tissue compared with normal tissue has been revealed in many human cancers^17–20^. Besides, Theodoropoulos et al. reported that HIF-1A was overexpressed in BCa, and its overexpression was related to poor prognosis^21^. Moreover, our qRT-PCR results using 20 pairs of BCa tissues also showed that HIF-1A is upregulated in BCa tissues. As a key regulator, HIF-1α target genes play major roles in critical aspects of tumor biology. As a well-known HIF-1α target gene, VEGF expression can be induced by HIF-1α in many cancers, which can enhance vascular permeability, stimulate angiogenesis, and increase local tissue oxygenation^22–24^. Furthermore, HIF-1α target genes can promote EMT, which include snail, transcription factor 3, and zinc finger E-box-binding homeobox 1 and 2 (ZFHX1A, and ZFHX1B)^25,26^. Gene-expression array demonstrate that more than 1,000 genes are transactivated by HIFs in response to hypoxia^27^, Manalo et al. reported that the PTGIS is an important target gene of HIF-1α and may be directly regulated by HIF-1α^28^.

PTGIS encodes prostaglandin synthase (prostaglandin I2 synthase), catalyzes the conversion of prostglandin H2 to prostacyclin (prostaglandin I2, PGI2)^29^. In cancers, many studies displayed that PGI2 can suppress proliferation and metastasis by activating peroxisome-proliferator-activated receptors (PPARs)^30–32^. Our microarray analysis (GEO accession No. GSE76211) using BCa tissues and normal bladder epithelium indicated that PTGIS is significantly downregulated in BCa tissues, and further studies shows that overexpression of HIF-1A in BCa cell line can decrease the expression of PTGIS by binding the hypoxia response element (HRE) of its promoter. Increasing evidences show that the methylation of CpG sites in promoter regions affects the regulation of gene expression by HIF-1α. It has been reported that methylation free HIF-1α Binding Site is required in several genes expression regulation by HIF-1α, such as MUC17, S100A4 and Erythropoietin^33–35^. Recent researches reported that PTGIS promoter is hypermethylated in colorectal cancer^36,37^, MethHc database (http://methhc.mbc.nctu.edu.tw/php/index.php) shows that PTGIS has a high DNA methylation levels in BCa. Therefore, we speculate that DNA methylation could be the reason that PTGIS expression is downregulated by HIF-1α but not upregulated by it in BCa cell line.

## Materials and methods

### Human bladder tissue samples

Bladder cancer and paracancerous tissue samples (*n* = 20) were obtained from patients after surgery at Zhongnan Hospital of Wuhan University, informed consent was collected from all subjects, and the histology diagnosis was confirmed pathologically by two pathologists independently. All the tissues were immediately stored in liquid nitrogen or fixed in 4% PFA after collection from the operation room. The informed consent was signed by all subjects. All specimens collection and treatment were carried out in accordance with the approved guidelines according to the Ethics Committee at Zhongnan Hospital of Wuhan University (approval number: 2015029).

### Plasmid construction

CIRBP cDNA (518 bp) and PTGIS cDNA (1502 bp) were polymerase chain reaction amplified from human BCa cell lines cDNA library and both cloned into *2xFIag-pcDNA3* empty vector, using CIRBP forward primer sense EcoR1 5′-CGGAATTCATGGCATCAGATGAAGGC-3′; CIRBP reverse primer sense Xho1 5′-AACTCGAGTTACTCGTTGTGTGTAGCGT-3′; PTGIS forward primer sense BamH1 5′-TAGGATCCATGGCTTGGGCCGCGCT-3′; PTGIS reverse primer sense EcoR5 5′-TCGATATCTCATGGGCGGATGCGGTAGCG-3′; The DNA sequence was verified by sequencing. *p3xFlag-CMV-14-HIF-1A* plasmid was a gift from Dr. Zhongqiang Guo (Department of Urology, Zhongnan Hospital of Wuhan University). *pGL4.10-PTGIS* promoter plasmid and *pGL4.10-PTGIS* promoter mut plasmid were purchased from Obio Technology Corp. Ltd. (Shanghai, China). Biotin-label mRNA transcripts were purchased from GenePharma biotech company (Shanghai, China).

### Cell culture, transfections, and stable cell lines selection

Human BCa cell lines 5637, UM-UC-3, T24 and human epithelial SV40 immortalized uroepithelium cell line SV-HUC-1 were obtained from the Stem Cell Bank, Chinese Academy of Sciences in Shanghai, China. Human BCa cell lines BIU-87 were purchased from the Procell Co., Ltd. in Wuhan, China. The BCa cell lines were identified by the China Centre for Type Culture Collection in Wuhan, China. 5637, T24, SV-HUC-1, BIU-87 Cells were cultured in RPMI-1640 medium (Gibco, China) and UM-UC-3 cells was cultured in DMEM high glucose medium (Gibco, Australia), with 10% fetal bovine serum (FBS) (Gibco, Australia) in 5% CO_2_ at 37 ℃.

For transfection, cells were transfected with plasmids or siRNA oligonucleotides using Lipofectamine 2000 transfection reagent according to the manufacture’s protocol. The sense sequence of *CIRBP-siRNA (siCIRBP)* / *CIRBP-shRNA* (*shCIRBP*) was 5′-CCAGAGAUCUCGGGGAUUUTT-3′, the sense sequence of *control-siRNA* (NC) / *control-shRNA* (NC) was 5′-UUCUCCGAACGUGUCACGUTT-3′, and the sense sequence of *HIF-1A-siRNA* (*si HIF-1A*) was 5′-GCCGCUCAAUUUAUGAAUATT-3′, the sense sequence of *PTGIS-siRNA (siPTGIS)* was 5′-GGCUGAAGAAUUACAACAUTT-3′.

For stable cell line selection, UM-UC-3 cells were infected with *lentiviral-CIRBP-shRNA* and *lentiviral-control-shRNA* (*LV-NC*) for 24 h, and treated by 5 μg/ml puromycin (Sigma, USA) for 7 days for further selection.

### Cell proliferation, clonogenic survival assay, and migration

Cell proliferation was performed by MTT assay. After transfection for 48 h, BCa cells were plated into 96-well plates (3000 cells per well) in 200 μl suspension medium (RPMI-1640 medium or DMEM high glucose medium) to grow for another 5 days. After indicated days, 20 μl MTT solution (5 mg/ml) was added into each well and incubated for 4 h at 37 °C. Absorbance at 490 m was measured by Microplate reader (Cat. #SpectraMax M2, Molecular Devices, USA).

For clonogenic survival assay, 48 h after transfection, distinct BCa cells were seeded in 6-well plates (1000 cells per well) and grew into colonies for approximately 14 days. The cells were washed gently twice with PBS and fixed in PBS with 4% freshly made paraformaldehyde for 30 min and then stained by crystal violet. The number of colonies was counted and statistically analyzed.

The transwell migration assay was conducted using 24-well plates transwell chamber system (Corning, USA) with 8.0 µm pore size. Briefly, 48 h after transfection, BCa cells were seeded (3 × 10^4 cells per chamber) in 200 µl serum-free medium in the upper transwell chamber (Corning, USA), while the lower chamber was filled with 600 µl medium containing 10% FBS. After incubation for 24 h at 37 °C, the cells on the upper chamber of filter were removed, and the cells on the lower chamber were fixed with 4% PFA and stained by 0.1% crystal violet. Then the chambers were placed under an inverted phase contrast microscope, the number of cells was counted and photographed in three random fields.

### RNA isolation and quantitative real-time PCR

Total RNA was isolated by HiPure Total RNA Mini Kit (Magen, China) from BCa cells and bladder tissues, quantity of isolated RNA was measured by NanoDrop. The reverse transcription reaction was performed with ReverTra Ace qPCR RT Kit (Toyobo, China). 1 µl of the resulting cDNAs were used as templetes for each reaction of the RT-PCR with iQTM SYBR® Green Supermix (Bio-Rad, USA) in a final volume of 20 µl. Primer sequences are listed in Supplementary Table S1. Fold enrichment was calculated with the −ΔΔCt method relative to GAPDH.

### Total protein isolation, Western blot, and immunofluorescence staining

BCa cells were washed with PBS for three times and lysed by RIPA buffer containing protease inhibitor and phosphatase inhibitor (Sigma-Aldrich, USA) for 30 min on ice. The cell lysates were then centrifuged at 13,000×*g* for 15 min. The supernatants were collected and protein concentration was determined by Bradford protein assay (Bio-Rad, Germany). The isolated total protein samples (25–30 μg) were separated by 6-15% SDSPAGE and subjected to Western blot analysis. The immunoreactive bands were visualized using an enhanced chemiluminescence kit (Bio-rad, USA) and detected by Molecular Imager Chemi Doc XRS^+^ Imaging system (Bio-rad, USA). The primary antibodies and secondary antibodies were listed in Supplementary Table S2. Immunofluorescence staining for BCa cells seeded on 12 mm coverslips and fixed tissue samples were accomplished by Biofavor Biotech company (Wuhan, China), immunofluorescence staining was analysed by using a Confocal microscope system (Nikon C2^+^ Confocal Microscope, Japan).

### mRNA stability assay, cycloheximide (CHX) assay, RNA-binding protein immunoprecipitation assay, and biotin pull-down assay

In order to examine the effects of CIRBP on the stability of the mRNAs of HIF-1A, 24 h after transfection, Cells were pretreated with CoCl_2_ (100 μM, Sigma c8661) for 4 h, then Actinomycin D (15 μg/ml, Abcam ab141058) was added to inhibit transcription. Total RNA was collected at specific time points of 0, 2, 4, 6, 8 h, and RT-PCR detected mRNA levels of HIF-1A.

In order to examine the effects of CIRPR on HIF-1α protein stability, 24 h post-transfection, Cells were pretreated with CoCl_2_ (100 μM) for 4 h, then cycloheximide (200 µg/ml, MCE, HY-12320) was added inhibit protein synthesis. Total protein samples were collected at specific time points of 0, 2, 4 h, and Western blot detected protein levels of HIF-1α.

For RNA-Binding Protein Immunoprecipitation (RIP) assay, 24 h after transfection with CIRBP overexpression plasmid, the cells on the plates were washed twice with 5 ml of ice-cold PBS, then lysed with1 ml lysis buffer (150 mM NaCl, 1% IGEPAL CA-603, 0.5% DOC, 0.1% SDS, 50 mM Tris 100 U/ml Rnasin, 1 mm PMSF) for 10 min, the cell lysates were then centrifuged at 16,000×*g* for 10 min and the supernatants were collected. 500 μl supernatants were incubated with 100 μl Protein G Sepharose beads (GE Healthcare Life Sciences, USA) with rotating for overnight which had been pre-coated with 40 μg of either anti-CIRBP or Normal rabbit anti-IgG1 for 6 h. The beads were washed five times (0.5 ml wash, 5 min each) with Wash buffer (50 mM Tris-HCl, 150 mM NaCl, 1 mM MgCl_2_, 0.05% NP-40 and 100 U/ml Rnasin), bound proteins were then digested by proteinase K Buffer (adding 0.1% SDS and 0.5 mg/ml proteinase K) at 55 °C for 30 min. Total RNA was isolated by chloroform extraction and resuspended in DEPC-treated water. RT-PCR analyzed the mRNA levels of HIF-1A, then the IP efficiency was calculated by percent of input (Percent Input = 2% x 2^(CT 2%Input Sample−CT IP Sample)^.

Biotin pull-down assays were performed by incubating 40 μg of cytoplasmic fractions with 50 pmol of biotin-label mRNA transcripts for 1 h at room temperature, and the ribonucleoprotein complexes were then isolated with 50 µl of streptavidin-conjugated Dynabeads (Invitrogen, # 11205D). The presence of CIRBP in the pull-down pellets was verified using Western blot analysis.

### Chromatin immunoprecipitation (ChIP) assay and luciferase reporter assay

The ChIP assay was performed with Simple CHIP® Plus sonication Chromation IP Kit (#56383, Cell Signaling Technology, USA) and carried out according to the manufacturer’s protocol. Briefly, 24 h after transfection with *p3xFlag-CMV-14-HIF-1A* plasmid or empty vector, cells were Cross-linked in 37% formaldehyde, and scraped into cold buffer, then continued with nuclei preparation and chromatin digestion. DNA fragment size was determined by electrophoresis on a 1% agarose gel (approximately 100–1000 bp). Then cross-linked chromatin was incubated with 2 μg anti-FLAG or normal rabbit IgG for overnight at 4 °C with rotation. After washing the protein G magnetic beads with low salt washing buffer and high salt washing buffer, Chromatin was eluted from Antibody/Protein G Magnetic Beads, detected by quantitative realtime PCR and calculate the IP efficiency (Percent Input = 2% x 2^(CT 2%Input Sample−CT IP Sample)^), Primer sequences for PTGIS promoter are listed in Supplementary Table S3.

For the luciferase reporter assay, cells were seeded onto 24-well plates and cotransfected with 500 ng of luciferase reporter plasmids (pGL4.10-PTGIS promoter vector, pGL4.10-PTGIS promoter mut vector or pGL4.10 base vector), and 500 ng of HIF-1A expression vector or empty vector. After 24 h, luciferase activity was measured with Luc-Pair ^TM^ Duo-Luciferase Assay Kit 2.0 (GeneCopoeial lnc, USA) according to its protocol.

### Xenograft model and pulmonary metastasis model

Male BALB/c-nu mice (4-weeks old) were purchased from Beijing Vital River Laboratory Animal Technology Co., Ltd. (Beijing, China), and maintained in laboratory animal facility of Zhongnan Hospital of Wuhan University. After adaptive feeding for a week, xenograft models were established by subcutaneously inoculating 1 × 10^6^ UM-UC-3 *LV-NC* cells or *LV-CIRBP sh* cells diluted in100 µl PBS (n = 4), while pulmonary metastasis models were established by tail intravenous injecting 1 × 10^6^ cells diluted in 100 µl PBS (*n* = 3). 6 weeks post injection, the mice were sacrificed and the tumors were taken out and weighed, meanwhile the tumor volume was measured every 6 days (tumor volume = length × width × 0.5 mm^3^). The fluorescence of pulmonary metastasis tumor was measured by FUSION FX7 Spectra Imaging system (Vilber, France).

### Statistical analyses

Data were expressed as mean ± SD from three individual experiments, statistical analyses were performed with SPSS Statistics 22.0. Unpaired 2-tailed *T*-test and one-way analysis of variance were used to evaluate statistical significance of differences of data, *p* < 0.05 were considered significant.

## Results

### CIRBP acts as an oncogene during BCa progression

**Fig. 1:**
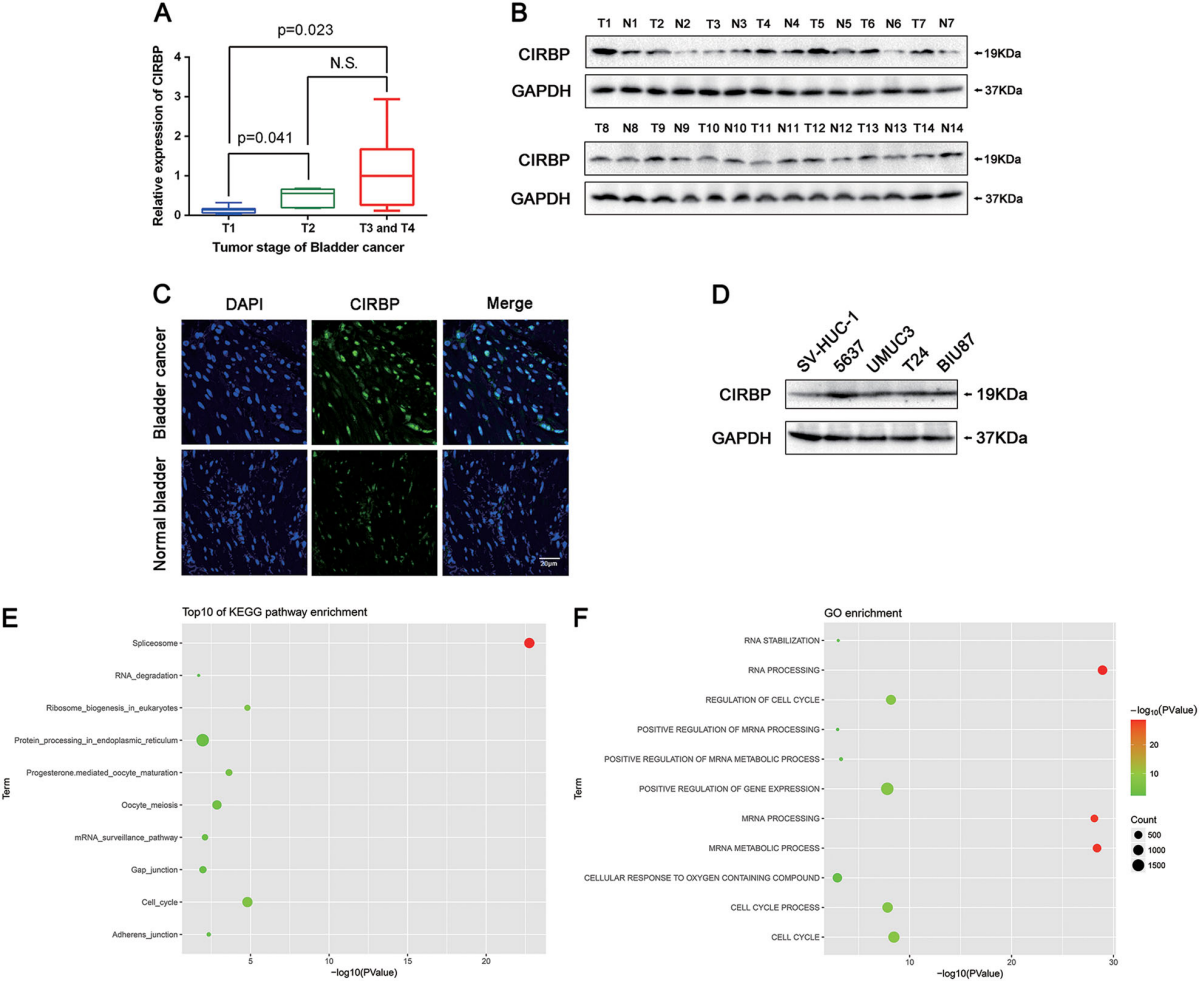
CIRBP protein is upregulated in 57% (8 of 14) BCa tissues and BCa cell lines, positively correlation between CIRBP expression levels and the T stage BCa, and pathway enrichment of CIRBP. **a** qRT-PCR analysis of CIRBP levels in different T stage BCa tissues. **b** Western blot analysis of the protein level of CIRBP in 14 pairs of BCa tissues and paracancerous tissues, GAPDH was used as a loading control. **c** Representative immunofluorescence staining of CIRBP (green) in the BCa tissue comparing with the normal bladder tissue, nuclei were stained by DAPI (blue). The images were photographed by Confocal microscope system. **d** Western blot analysis of CIRBP protein abundance in four BCa cell lines (5637, UM-UC-3, T24 and BIU-87) and immortalized normal uroepithelial cell line (SV-HUC-1). **e** and **f** KEGG pathway enrichment and GO enrichment of CIRBP in miRNA Cancer MAP database

To investigate the CIRBP expression level in BCa, CIRBP mRNA was analyzed by qRT-PCR analysis, indicating no significant difference in the BCa tissues compared with the paired paracancerous tissues (*n* = 21, Supplementary Figure S1A). We next analyzed the association between the CIRBP mRNA levels and the tumor stage of these BCa tissues, indicating that CIRBP expression levels were positively correlated with the T stage in BCa (Fig. 1a). Furthermore, Western blot analyzed the protein level of CIRBP in 14 pairs of BCa tissues and paracancerous tissues, CIRBP protein upregulation was observed in 8 of 14 (57%) BCa tissues (Fig. 1b). To further confirm CIRBP protein upregulation, immunofluorescence staining was performed, CIRBP was observed in the nucleus, and it revealed strong increase of CIRBP protein in the BCa tissues (Fig. 1c). In addition, we detected the CIRBP protein levels in four BCa cell lines (5637, UM-UC-3, T24 and BIU-87) and immortalized normal uroepithelial cell line (SV-HUC-1), exhibiting an upregulation tendency of CIRBP in BCa cell lines (Fig. 1d). To obtain further insight into the function of CIRBP, we searched the gene function enrichment by using miRNA Cancer MAP database (http://cis.hku.hk/miRNACancerMAP/index.php). As shown in Fig. 1e, f, CIRBP was significantly enriched in several pathways, such as RNA degradation, cell cycle, adherens junction.

**Fig. 2:**
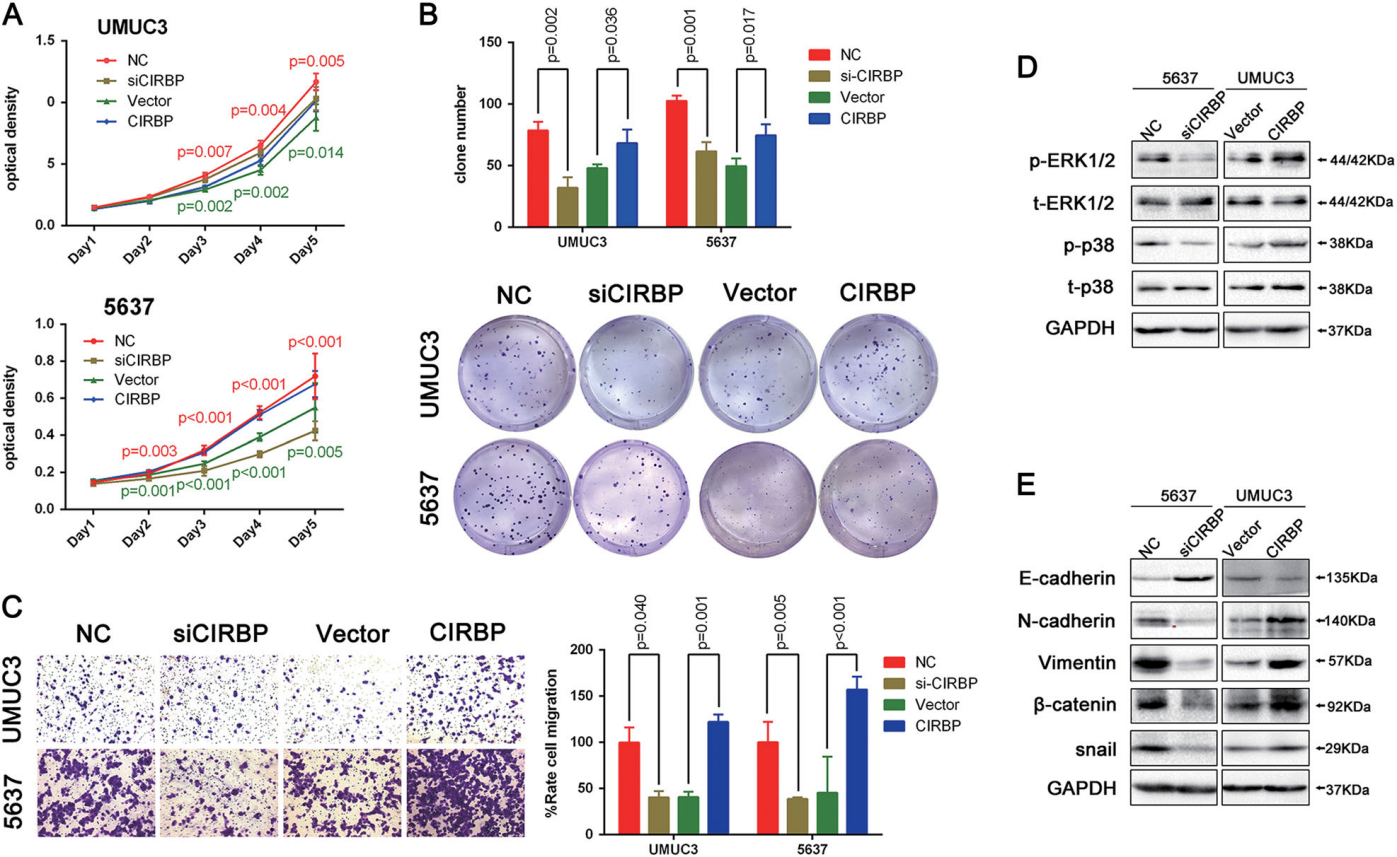
CIRBP promotes BCa cells proliferation and migration in vitro, increases the phosphorylation state of ERK1/2 and p38, also promotes the EMT in BCa cells. **a** Distinct BCa cells (UM-UC-3 and 5637) transfected with NC (red), *siCIRBP* (brown), vector (green) or CIRBP overexpression plasmid (blue) were allowed to grow at the indicated times, and Cell growth and viability were evaluated by MTT assay. **b** Clonogenic survival assay evaluated Colony formation, and statistical analysis of the clone number. **c** Cell migration was evaluated by transwell migration assay, and the relative cell number of migration was statistically analyzed. **d** Proteins in the MAPK family, including phosphorylated and total ERK1/2 and p38, were analyzed by Western blot, suggesting activation of ERK1/2 and p38 by CIRBP in BCa cells. **e** Western blot analyzed the proteins (E-cadherin, N-cadherin, Vimentin, β -catenin and snail) involved in EMT after 48 h transfection of *siCIRBP* in 5637 cells or CIRBP overexpression plasmid in UM-UC-3 cells. Means ± standard deviation from three independent experiments. Student *t* test was used for statistical analysis

To study the effects of CIRBP on the biological behaviors of BCa cells, an siRNA specifically targeting CIRBP was used to knock down CIRBP expression, while an CIRBP gene overexpression plasmid was used to upregulate CIRBP expression in two BCa cell lines (UM-UC-3 and 5637). Western blot analysis verified the overexpression and knockdown efficiencies, and qRT-PCR analysis demonstrated the same results in mRNA levels (Supplementary Figure S1B). Next, we evaluated the cell proliferation and migration abilities. MTT assay showed that *CIRBP-siRNA*-treated BCa cells grew significantly slower than the control BCa cells, conversely, overexpression of CIRBP dramatically promoted cell growth (Fig. 2a). In agreement with its facilitated proliferation function, clonogenic survival assay revealed that the ability of colony formation was significantly decreased in the CIRBP knockdown cells, while increased in CIRBP overexpression cells (Fig. 2b). What’s more, transwell migration assay suggested that knockdown of CIRBP in BCa cells could reduce cell migration and overexpression of CIRBP could induce cell migration (Fig. 2c).

### Effects of CIRBP on MAPK signaling pathway and EMT markers

MAPK pathway plays an important role in various tumor physiological processes, including cell proliferation, cell differentiation and cell survival^38,39^, our transcriptome data also indicated that MAPK signaling pathways play a key role in BCa^40,41^. CIRBP has been reported to be involved in multiple tumor progression via the activation of ERK and p38 MAPK^7,42,43^. Our Western blot analysis showed phosphorylated ERK1/2 (p-ERK1/2) and phosphorylated p38 (p-p38) protein levels were obviously decreased in the *si-CIRBP*-treated 5637 cells, whereas increased in the CIRBP overexpression UM-UC-3 cells (Fig. 2d).

Moreover, because CIRBP could promote cell migration, and pathway enrichment indicated that CIRBP is related with adherens junction, proteins involved in the EMT process were analyzed by Western blot analysis. CIRBP knockdown could suppress the expression of N-cadherin, vimentin, β-catenin and snail in 5637 cells, reversely, CIRBP overexpression could increase their expression level in UM-UC-3 cells. In addition, knockdown of CIRBP could upregulate the epithelial marker E-cadherin in 5637 cells, and overexpression of CIRBP could reduce E-cadherin protein expression in UM-UC-3 cells (Fig. 2e).

### Reduction of CIRBP inhibits BCa growth and pulmonary metastasis in vivo

**Fig. 3:**
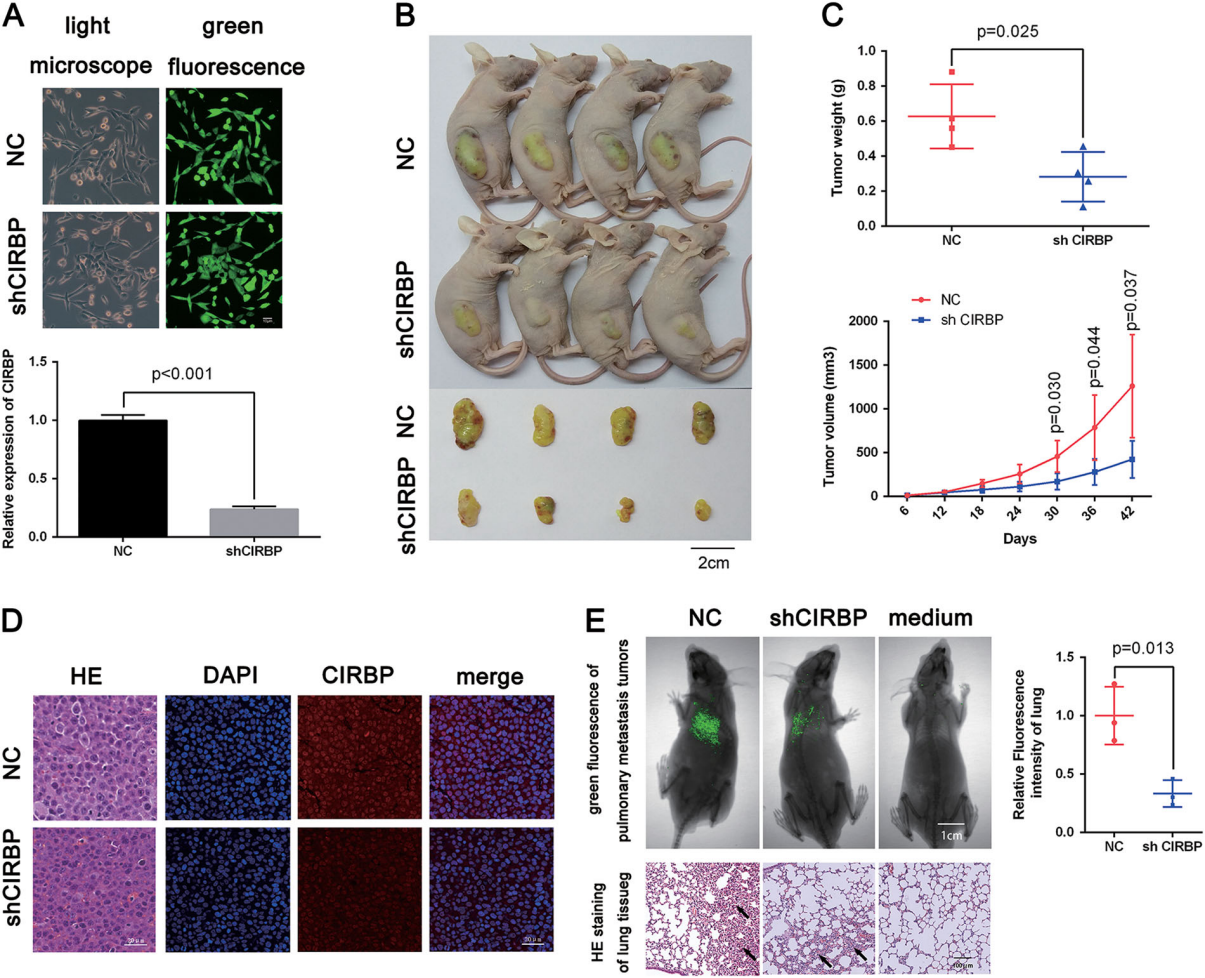
Knockdown of CIRBP inhibits BCa growth and migration in vivo. **a** The green fluorescence of stable cell lines, and qRT-PCR analysis verification of CIRBP knockdown efficiency by *lentiviral-CIRBP-shRNA*. **b** Xenograft models (*n* = 4) were established by subcutaneously inoculating *LV-NC* cells or *LV-shCIRBP* cells and allowed to grow for 6 weeks, then the mice were sacrificed and the tumors were taken out and weighed. **c** tumor volume measurement and tumor weight. **d** Representative H&E staining and immunefluorescence staining of xenograft tumors from the tumor-bearing mice of the *LV-NC* group and *LV-shCIRBP* group, indicating the downregulation of CIRBP (red) in *LV-shCIRBP* group. Nuclei were stained by DAPI (blue). **e** Pulmonary metastasis models (*n* = 3) were established by tail intravenous injecting *LV-shCIRBP* UM-UC-3 cells or *LV-NC* UM-UC-3 cells, the fluorescence intensity of pulmonary metastasis tumor was measured to evaluate the migration capacity. Representative H&E staining of lung tissues indicating the pulmonary metastasis tumors (pointed by the arrows). Statistical analysis of the fluorescence intensity was calculated using *T*-test. Means ± standard deviation from three independent experiments

To verify the function of CIRBP in vivo, *lentiviral-CIRBP-shRNA* and *lentiviral-control-shRNA* (*LV-NC*) stable cell line were established, after selection with puromycin for 7 days, the green fluorescence of stable cell lines was showed in Fig. 3a, qRT-PCR analysis validated the Efficiency of CIRBP knockdown by *lentiviral-CIRBP-shRNA* (Fig. 3a). Next, as shown in Fig. 3b, c, xenograft models were construct, the *LV-shCIRBP* group grew significantly slower than the control group in vivo, and the mean tumor weight was significantly lower in the *LV-shCIRBP* group than in the *LV-NC* group (Fig. 3c). The dissected neoplasms tissues were embedded into paraffin, the Hematoxylin and Eosin staining (HE staining) results suggested the reduced number of tumor cells in the *LV-shCIRBP* group, and the immune-fluorescence staining displayed that CIRBP expression is weaker in the *LV-shCIRBP* group compared with the *LV-NC* group (Fig. 3d).

In the same way, for the purpose of investigating the effect of CIRBP knockdown on BCa metastasis in vivo, pulmonary metastasis models were established by tail intravenous injecting *LV-shCIRBP* UM-UC-3 cells or *LV-NC* UM-UC-3 cells, after 4 weeks of injection, the fluorescence of pulmonary metastasis tumor was measured to evaluate the migration capacity. As exhibited in the Fig. 3e, CIRBP knockdown could significantly suppress migration in vivo in contrast with the *LV-NC* group, the HE staining displayed the pulmonary metastasis tumors (pointed by the arrows).

### CIRBP increases mRNA stability and protein translation of HIF-1α in BCa cell line

**Fig. 4:**
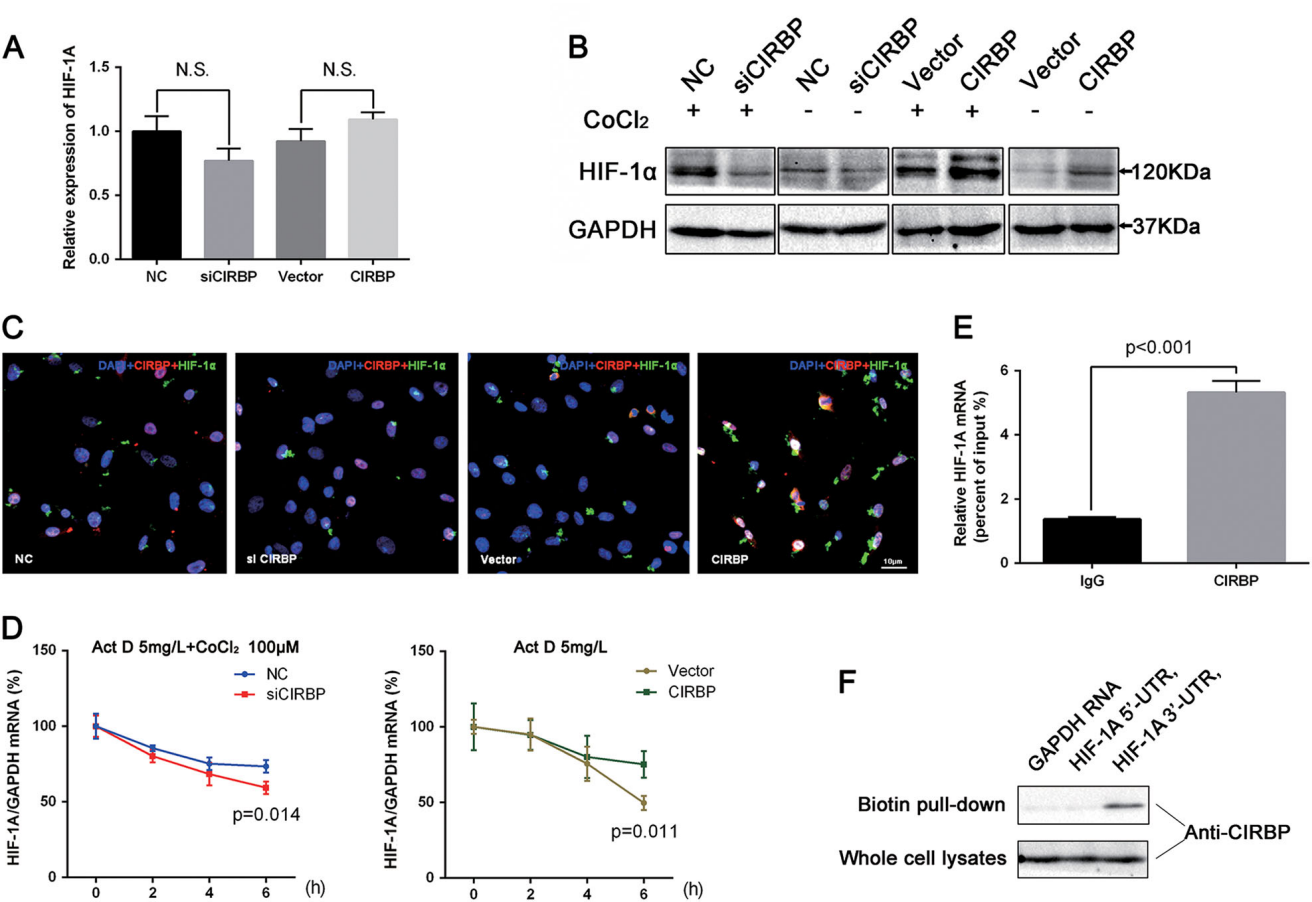
CIRBP increases mRNA stability and protein translation of HIF-1α in BCa cells. **a** qRT-PCR analysis for the effects on the HIF-1A mRNA levels of CIRBP knockdown and CIRBP overexpression. **b** Western blot analysis of HIF-1α performed on CIRBP knockdown and CIRBP overexpression UM-UC-3 cells, exposed (+) or not (−) to CoCl_2_ (100 μM for 4 h, Sigma 15862). **c** Immunofluorescence staining revealed alterations of HIF-1α (green) after 48 h transfection of *siCIRBP* in UM-UC-3 cells or CIRBP overexpression plasmid in UM-UC-3 cells under normoxia (CIRBP red). Nuclei were stained by DAPI (blue). **d** 24 h after transfection with *siCIRBP* (pretreated with CoCl_2_) or CIRBP overexpression plasmid (under normoxia), UM-UC-3 cells were cultured in the presence of Actinomycin D (15 μg/ml, Abcam ab141058), qRT-PCR detected mRNA levels of HIF-1A specific time points of 0, 2, 4, 6 h. GAPDH is used as the normalization control. **e** RNA-Binding Protein Immunoprecipitation (RIP) assay was performed with anti-CIRBP or anti-IgG, qRT-PCR detected the IP efficiency (percent input). **f** Biotin pull-down assay was achieved to confirm the interacts of CIRBP and *HIF-1A* mRNAs 3′-UTR, both 3′-UTR and 5′-UTR of *HIF-1A* mRNA transcripts were constructed with biotin-label, transcripts of GAPDH mRNA were used as a negative control. Means ± standard deviation from three independent experiments. *T*-test was used for statistical analysis

To investigate the relationship between CIRBP and HIF-1α in BCa, we first evaluated whether HIF-1α could modulate the expression of CIRBP, qRT-PCR analysis showed that HIF-1A overexpression have no obvious effect on the mRNA level of CIRBP (Supplementary Figure S1C). Next, the study addressed the effects of CIRBP on HIF-1α expression, as shown in the Fig. 4a, qRT-PCR analysis showed that both CIRBP knockdown and CIRBP overexpression had no significant effects on the mRNA levels of HIF-1A. Moreover, Western blot analysis showed that transfection of *si-CIRBP* could robustly downregulate HIF-1α expression, and overexpression of CIRBP could significantly upregulate HIF-1α expression, both under normoxia and hypoxia (Fig. 4b), the same results were also revealed by immunofluorescence staining under normoxia (Fig. 4c).

UniGene database indicated that HIF-1A is one of CIRBP-targeted transcripts^15^, and pathway enrichment indicated that CIRBP was significantly enriched in RNA degradation and RNA stabilization. To determine whether CIRBP upregulate HIF-1α expression through binding to the 3′-UTR and increases its mRNA stability, we first performed mRNA stability experiments in the presence of actinomycin D (a transcriptional inhibitor, 15 μg/ml), as displayed in Fig. 4d, under the hypoxia conditions (pretreated with CoCl_2_ 100 µM for 4 h), the results demonstrated that transfection with *si-CIRBP* reduced the stability of HIF-1A mRNA. While in absence of CoCl_2_, CIRBP overexpression could increase HIF-1A mRNA stability strikingly. Next, RNA-Binding Protein Immunoprecipitation (RIP) assay was used to demonstrate that CIRBP could bind to HIF-1A mRNA (Fig. 4e). Furthermore, biotin pull-down assay was achieved to confirm the interacts of CIRBP and HIF-1A mRNAs 3′-UTR, as shown in Fig. 4f, substantial amounts of CIRBP were associated with the HIF-1A mRNA transcripts 3’-UTR, nevertheless, both the 5’-UTR of HIF-1A mRNA transcripts and the negative control transcripts failed to pull down CIRBP. In addition, in order to investigate the effects of CIRBP on HIF-1α protein stability, cycloheximide (CHX) assay was performed. As shown in Supplementary Figure S1D, CIRBP knockdown had no obvious effects on the HIF-1α protein degradation.

### PTGIS is a HIF-1α target gene in BCa

**Fig. 5:**
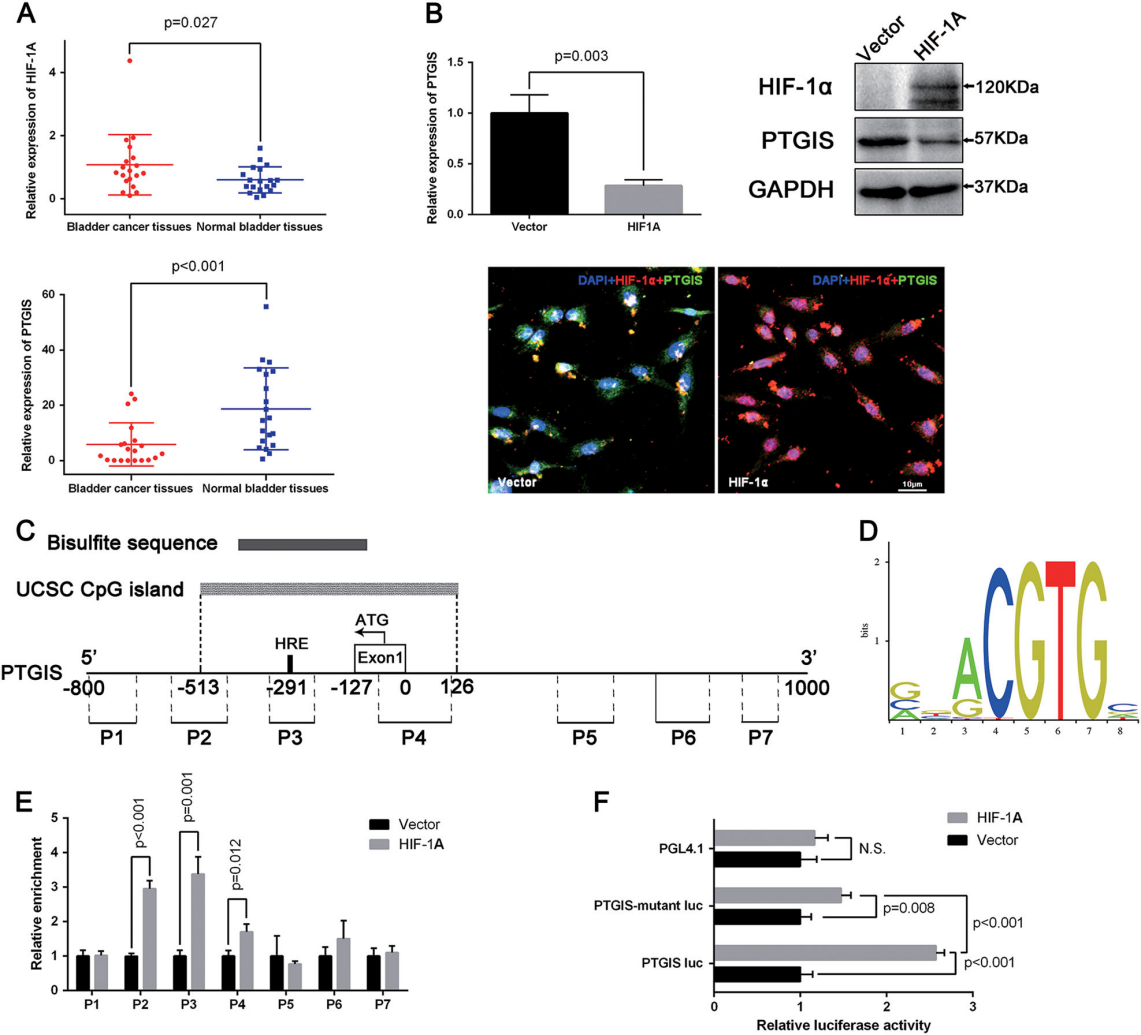
HIF-1α suppresses the expression of PTGIS in BCa cells by binding to HRE sequence of the PTGIS promoter region. **a** qRT-PCR analysis exhibited the expression of HIF-1A and PTGIS at the transcription level in BCa tissues compared with paracancerous tissues (*n* = 20). **b** qRT-PCR analysis, Western blot analysis and immunofluorescence staining (HIF-1α: red, PTGIS: green) for the effects on the PTGIS expression levels of HIF-1A overexpression. **c** The schematic representation of the PTGIS promoter region. HRE (5′-ccACGTGc-3′, −292 to −285), Exon 1 (−127 to 0), and translation start site (ATG, −72) are indicated on the genomic PTGIS sequence. The gray box represents the UCSC CpG island (−513 to 126) of PTGIS promotor region, the black box represents the bisulfite sequence region (−422 to −166) of CpG island. The thick truncated lines (P1-P7) mark the regions covered by primer sets of CHIP PCR. **d** The binding site of HIF-1α provided by the JASPAR database. **e** The ChIP assay was conducted to analyze the local enrichment of FLAG-tagged HIF-1α across the PTGIS promote region in FLAG-tagged HIF-1A transfected UMUC3 cells compared with empty vector transfected UMUC3 cells. The relative fold enrichment was quantified by normalization to input first, then normalized to empty vector transfected UMUC3 cells, which is set at 1. **f** Luciferase assay in UM-UC-3 cells co-transfected with PTGIS luciferase reporter plasmids (*pGL4.10-PTGIS* luciferase vector, *pGL4.10-PTGIS HRE mutant* luciferase vector or *pGL4.10* base vector), and HIF-1A overexpression plasmid or empty vector. Values represent the mean ± SD of three independent experiments

As a master regulator in hypoxic cancer progression, HIF-1α activates transcription of many genes^23^, two publications of microarray hybridization analysis have reported that PTGIS is an important target gene of HIF-1α^28,44^, and our microarray analysis (GEO accession no. GSE76211) indicate that PTGIS is a significantly downregulated gene in BCa tissues compared with normal bladder tissues. Therefore, our following studies focused on the regulatory effects of HIF-1α on PTGIS expression in BCa. In the same way, we first detected the mRNA levels of HIF-1A and PTGIS in the BCa tissues (n = 20), the results of qRT-PCR analysis displayed that HIF-1A is significantly upregulated, while PTGIS is remarkably downregulated in the BCa tissues (Fig. 5a). Furthermore, to detect the transcription effects of HIF-1α on PTGIS expression, a HIF-1A gene overexpression plasmid was used, as shown in Fig. 5b, overexpression of HIF-1α could suppress the expression of PTGIS both in mRNA level and protein level, immunofluorescence staining also proved the same conclusion. In addition, *HIF-1A* knockdown with a siRNA specifically targeting *HIF-1A* could increase the PTGIS mRNA level (Supplementary Figure S1E), the overexpression and knockdown efficiencies were validated by qRT-PCR (Supplementary Figure S1E).

As a key transcription factor of the adaptive response to hypoxia, HIF-1α transcriptional regulates many genes through binding to core DNA sequence 5’-RCGTG-3’ within the HRE of target gene promoters^45^. We next predicted an HRE binding site (5′-ccACGTGc-3′) of HIF-1α on PTGIS promoter region through using GCBI database (https://www.gcbi.com.cn/gclib/html/index), in addition, as shown in Fig. 5d, the binding site of HIF-1α provided by the JASPAR database (http://jaspardev.genereg.net/) supported this conclusion. Furthermore, a ChIP assay was performed to verify this conclusion, after transfection for 24 h, DNA was digested with ultraphonic to a length of approximately 100-1000 bp (Supplementary Figure S1F), and the anti-FLAG was employed to pull down HIF-1α and its associated DNA fragments. As shown in Fig. 5c, seven pairs of primers were designed to cover the region of the PTGIS promoter, qRT-PCR detected the enrichment results. And the highest enrichment of *FLAG-HIF-1A* was identified at the P3 region of the PTGIS promoter, followed by the P2 and P4 region, while no significant enrichment was found in other regions (Fig. 5e). Subsequently, luciferase reporter assay was used to evaluate the effects of HIF-1α on PTGIS promoter activity, as shown in Fig. 5f, overexpression of HIF-1A could obviously enhance the luciferase activity of PTGIS luc compared with empty vector, while mutant of HRE (5′-ccTGCACc-3′) sequence could significantly reduce this induction.

### CpG Methylation of PTGIS promoter region plays an important role in transcriptional regulation of HIF-1α

**Fig. 6:**
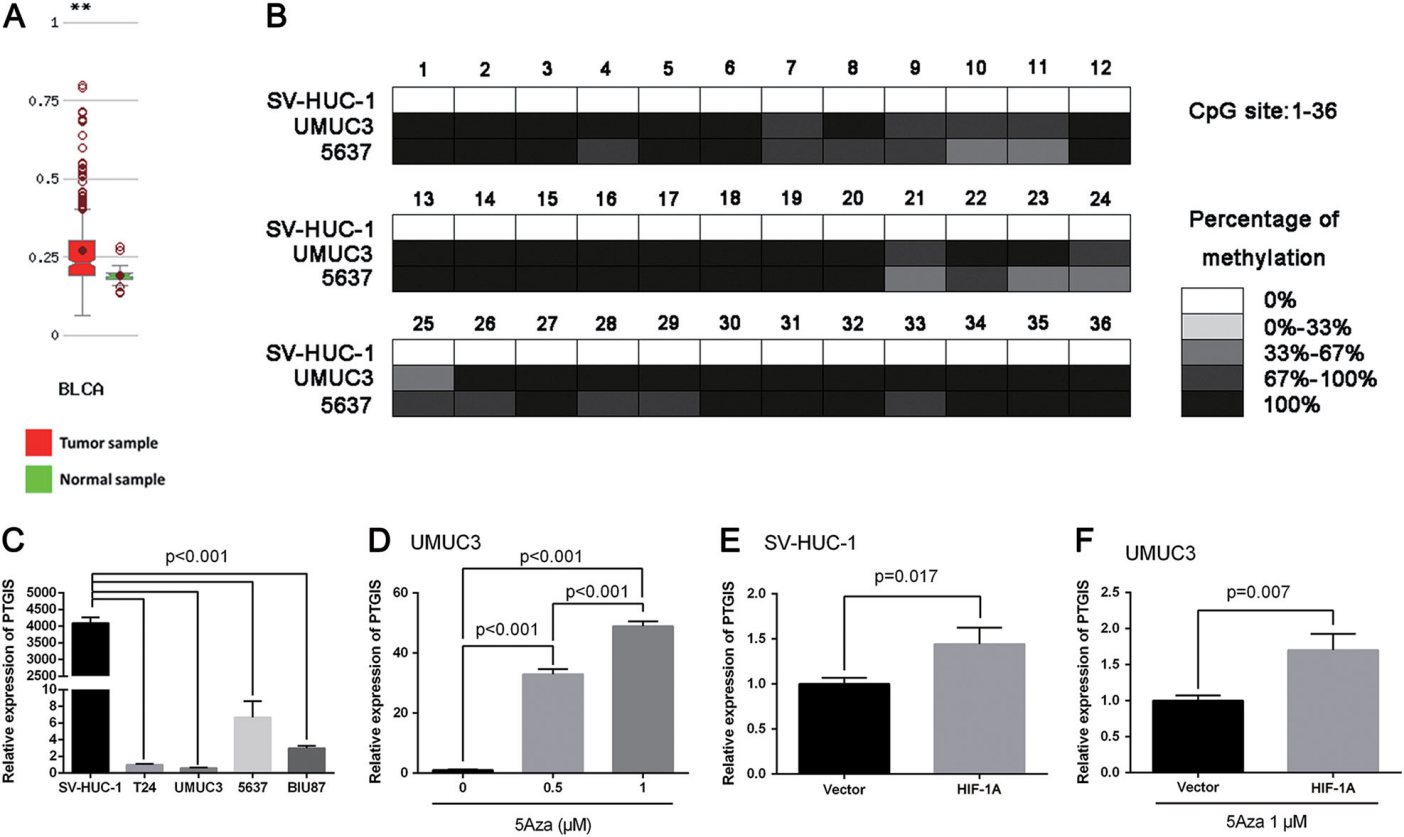
CpG Methylation of PTGIS promoter region plays an important role in transcriptional regulation of HIF-1α. **a** MethHc database showed that PTGIS has a high DNA methylation levels in BCa compared with normal samples. **b** Bisulfite sequence analyses of 36 CpG sits of PTGIS promotor region in two BCa cell lines (UM-UC-3 and 5637) and immortalized normal uroepithelial cell line (SV-HUC-1). **c** qRT-PCR analysis detected the mRNA level of PTGIS in distinct malignancy BCa cells (T24, UM-UC-3, 5637, BIU-87) and immortalized normal uroepithelial cell line (SV-HUC-1). **d** qRT-PCR analysis detected the mRNA level of PTGIS in UM-UC-3 cells after the treatment by the demethylating agent 5-Aza (Cayman Chemical #11166-5) at 0, 0.5 and 1 μM for 24 h. **e** qRT-PCR analysis detected the effects of HIF-1A overexpression on PTGIS in SV-HUC-1 cells. **f** qRT-PCR analysis detected the effects of HIF-1A overexpression on PTGIS in demethylating UM-UC-3 cells (pre-treatment with 1 µM 5Aza for 24 h)

CpG methylation plays an important regulatory role in the control of gene expression^46^, several researches had reported that the promoter regions methylation affects the regulation of gene expression by HIF-1α^33,34,47^. HIF-1α could activate transcription of many target genes, especially many oncogenes such as VEGF, erythropoietin, C-MYC and OCT4^23,45^. However, our results revealed that overexpression of HIF-1α can suppress the expression of PTGIS in UM-UC-3 cells, so our following studies focused on the relationship between CpG Methylation of PTGIS promoter region and transcriptional regulation of HIF-1α on PTGIS. As shown in Fig. 6a, MethHc database showed that PTGIS has a high DNA methylation levels in BCa compared with normal samples, and its DNA methylation was negatively correlated with PTGIS mRNA expression levels in BCa (Supplementary Figure S1G). In addition, UCSC Genome Browser database (http://genome.ucsc.edu/) indicated that PTGIS gene promoter region has a CpG island (Position: chr20: 49567657-49568296), which was displayed in Fig. 5c.

In order to confirm the methylation of PTGIS gene promoter in BCa cell lines, Bisulfite sequence analyses were performed in three cell lines (SV-HUC-1, UM-UC-3 and 5637), demonstrating that methylation of all CpG sites of PTGIS gene promoter is more frequent in BCa cell lines (UM-UC-3 and 5637) rather than immortalized normal uroepithelial cell line (SV-HUC-1, Fig. 6b). Apart from downregulation in the BCa tissues, our qRT-PCR results indicated that PTGIS expression is strikingly reduced in BCa cell lines compared with SV-HUC-1 (Fig. 6c). Next, to further prove the methylation of PTGIS, UM-UC-3 cells were cultured with the demethylating agent 5-Aza-2’-deoxycytidine, and qRT-PCR detected the restoration of PTGIS expression in BCa cells (Fig. 6d). Then we investigated the effects of *HIF-1A* overexpression on PTGIS in SV-HUC-1 cells, which have a low PTGIS gene promoter methylation level. As shown in Fig. 6e, in contrast with transcriptional suppression in UM-UC-3 cells, transfection of *HIF-1A* overexpression plasmids could upregulate the expression level of PTGIS. Besides, when UM-UC-3 cells were pre-treated with 5Aza (1 µM for 24 h), the previous transcriptional suppression would turn to transcriptional activation (Fig. 6f).

### PTGIS acts as a tumor suppressor in BCa

**Fig. 7:**
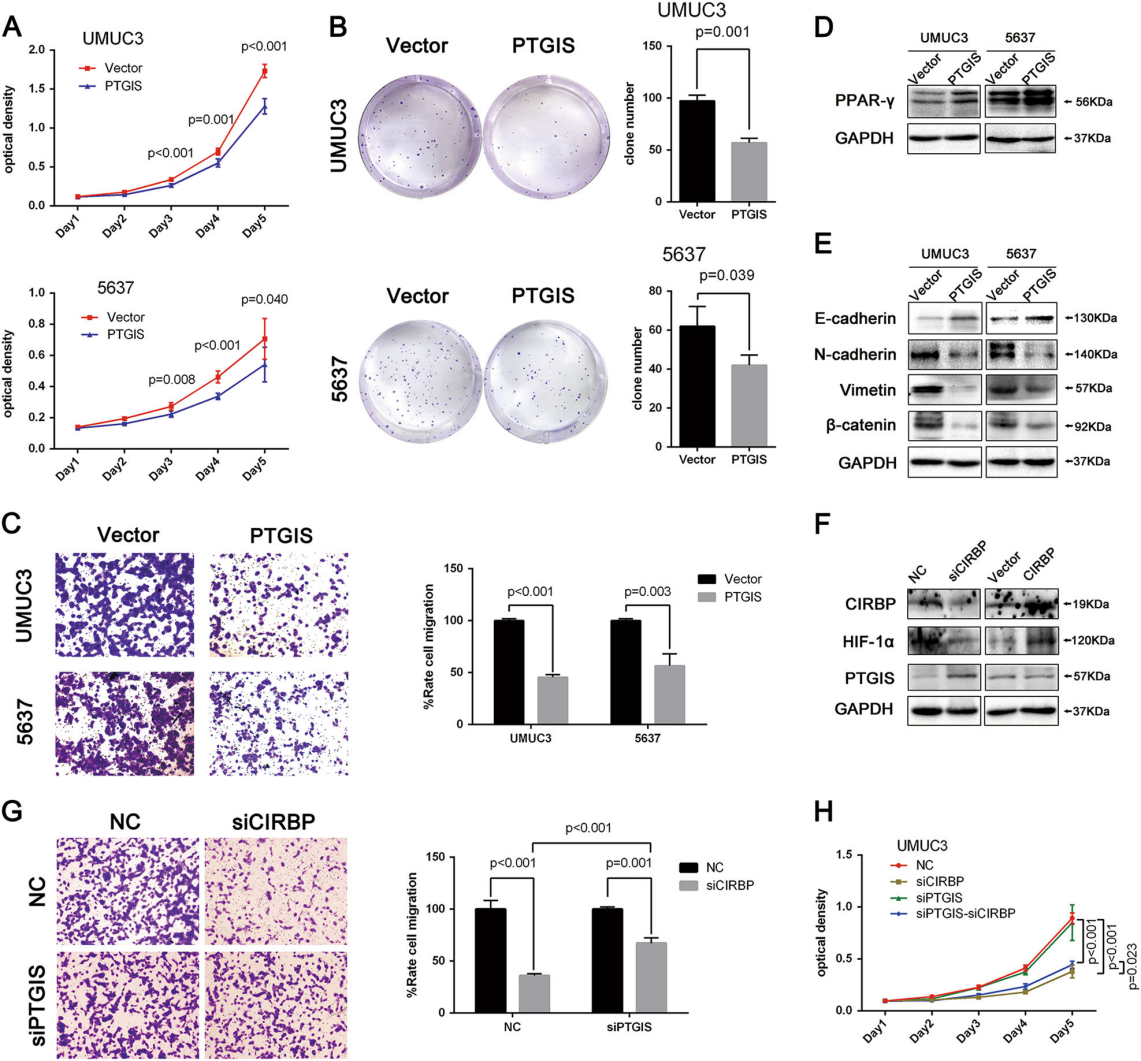
Overexpression of PTGIS suppresses BCa cells proliferation and migration in vitro, increases the protein level of PPARγ and suppresses EMT. PTGIS knockdown could partially rescue the inhibition of migration and proliferation in *siCIRBP*-treated BCa cells. **a** After transfection with PTGIS overexpression plasmid, cell proliferation was performed by MTT assay. **b** Clonogenic survival assay and statistical analysis of the clone number. **c** Transwell migration assay and the statistical analysis of relative cell number of migration. **d**, **e** Western blot analysis of PPARγ and EMT markers (E-cadherin, N-cadherin, Vimentin and β -catenin) after 48 h transfection of PTGIS overexpression plasmid. **f** Western blot analysis of CIRBP, HIF-1α and PTGIS expression in *siCIRBP*-treated cells as well as in cells over-expressing CIRBP. **g**, **h** Rescue experiment of *siPTGIS*: Transwell migration assay and MTT assay

PTGIS has been reported to be a tumor suppressor in many kinds of cancers^30–32^, our results displayed that PTGIS is downregulated both in BCa tissues (Fig. 5a) and in BCa cell lines (Fig. 6c). Similarly, to explore the tumor biological functions of PTGIS in BCa, a PTGIS expression plasmid was constructed, and the overexpression efficiency was verified by Western blot (Supplementary Figure S1H). As shown in Fig. 7a, b, MTT assay and clonogenic survival assay demonstrated that overexpression of PTGIS could suppress BCa cells proliferation Moreover, the protein level of PPAR-γ was increased by transfection of PTGIS expression plasmids (Fig. 7d). In addition, transwell migration assay showed that PTGIS overexpression could significantly suppress migration of BCa cells (Fig. 7c), while Western blot results further proved that PTGIS overexpression could suppress the EMT in UM-UC-3 and 5637 cell lines (Fig. 7e).

### PTGIS knockdown could partially rescue the inhibition of migration and proliferation in siCIRBP treated BCa cells

Since CIRBP could induce expression of HIF-1α, and HIF-1α could suppress the expression of PTGIS in BCa cells, we finally explored the regulation relationship of CIRBP on PTGIS expression. As shown in Fig. 7f, knockdown of CIRBP could promote the expression of PTGIS, while overexpression of CIRBP have no obvious suppression effect on PTGIS expression, which may be associated with its low expression in BCa cells, and qRT-PCR analysis demonstrated the same results in mRNA levels (Supplementary Figure S1I). In addition, further studies showed that PTGIS knockdown could significantly rescue the inhibition of migration in siCIRBP treated BCa cells (Fig. 7g), and partial rescue of proliferation inhibition was also observed (Fig. 7h), while PTGIS inhibition have no effect on siCIRBP induced inhibition of clonogenic survival ability in BCa cells (Supplementary Figure S1J). The knockdown efficiency of siPTGIS was validated by Western blot (Supplementary Figure S1K).

## Discussion

Recently, CIRBP has been reported to act as an oncogene in many studies^9,10,43,48^, in this study, we investigated the involvement of CIRBP in BCa. Our results indicated that CIRBP is overexpressed in 57% BCa tissues and BCa cell lines, and CIRBP could promote BCa cells proliferation and migration both in vitro and in vivo. Furthermore, we demonstrated that CIRBP expression led to an increase in the phosphorylation state of ERK1/2 and p38, also promoted the EMT in BCa cells. MAPKs plays a central role in cell proliferation control^49^, our transcriptome data also indicated that MAPK signaling pathways play a key role in BCa^40,41^, in agreement with our results, evidence of a possible linkage between CIRBP and MAPK signaling pathways comes from two studies, Artero-Castro *et al*. reported that CIRBP displayed the ability to bypass replicative senescence through activation of the ERK1/2 signaling pathway in primary mouse embryonic fibroblasts^7^, the second study by Lee et al. showed that CIRBP could promote epithelial to mesenchymal transition by activating ERK and p38 pathways^43^.

RNA-binding proteins play an essential role in RNA metabolism^50^, CIRBP has been reported to regulate several target genes by specifically binding to the 3′-UTR of its target mRNAs. For instance, CIRBP could bind to the TRX 3′-UTR and stabilize the transcript to increase its translation^16^, another study showed that CIRBP could increase the stability of pro-inflammatory cytokine mRNAs under cold conditions to induce an airway inflammatory response^11^. The results of our study showed that CIRBP could induce HIF-1α protein production via a CIRBP-mediated increase in mRNA stability in BCa, and the same results had been reported in Melanoma LOX-IMVI cells in a study by Chang *et al*^51^. Combined with the RIP and biotin pull-down assay results, we calculate that this induction was through the way of specifically binding to the 3′-UTR of HIF-1A mRNA transcripts. HIF-1α plays a critical role in regulation of gene transcription as a response to hypoxia, several key regulatory steps participate in the mechanism by which changes in oxygen concentration can be directly transduced into changes in gene expression by HIF-1α^52^. Under normoxic conditions, HIF-1α proteins are rapidly ubiquitylated and degraded by the von Hippel-Lindau, and this process depends on the availability of O_2_^53^, besides, HIF-1α transcriptional activity is suppressed under normoxic conditions by hydroxylation of an asparagine residue within its C-terminal transactivation domain, which is mediated by factor-inhibiting HIF1 (FIH1)^54^. Our results also showed that CIRBP could increase mRNA stability and protein translation of HIF-1α in BCa cell line, this may be part of reason why HIF-1α could be induced under hypoxia conditions.

HIF-1α dependent regulations in gene expression usually involve the activation of transcription, especially activate many oncogenes in cancers^23,52^, two publications have reported that PTGIS may be an important target gene of HIF-1α^28,44^. Our results firstly indicated that HIF-1α could transcriptionally suppress the expression of PTGIS in BCa cells through binding to HRE sequence of the PTGIS promoter region. Further studies demonstrated that PTGIS promoter is hypermethylated in BCa, what’s more, overexpression of HIF-1A could increase the expression of PTGIS in SV-HUC-1 cells, which have a low PTGIS gene promoter methylation level. Similarly, after culturing with the demethylating agent, the previous transcriptional suppression would turn to transcriptional activation in BCa cells. Our research clarified that methylation of PTGIS gene promoter may be an important reason why PTGIS expression is downregulated by HIF-1α but not upregulated by it in BCa cell line. A few studies have reported the transcriptional suppression of HIF-1α on target genes promoter activity. A study by Chen *et al*. showed that HIF-1α transcriptionally suppressed cad-gene expression under hypoxia^55^, another study by Mazure et al. displayed that the existence of a possible competition between HIF-1α and c-Myc that could modulate the transcriptional activity of the AFP gene in response to hypoxia, which resulted in a suppression effect of hypoxia on AFP gene expression^56^. More researches are needed to explore the detailed mechanism for transcriptional suppression of HIF-1α on PTGIS expression in BCa.

Many studies have reported that PTGIS acted as a tumor suppressor in several cancers^30–32^, our microarray analysis (GEO accession No. GSE76211) revealed that PTGIS is significantly downregulated in BCa tissues, and qRT-PCR analysis results proved this conclusion. In addition, we proved that PTGIS overexpression could suppress the BCa cells proliferation and migration, together with our demonstration that PTGIS increased the protein level of PPAR-γ and suppressed EMT, we demonstrated that PTGIS also acts as a tumor suppressor in BCa. MethHc database showed that PTGIS promotor methylation was negatively correlated with PTGIS mRNA expression levels in BCa, this indicated that DNA methylation is an important reason for obvious downregulation of PTGIS in BCa. In contrast, GEPIA database (http://gepia.cancer-pku.cn/) showed no correlation between HIF-1A and PTGIS expression (Supplementary Figure S1L), indicating that HIF-1A upregulation in BCa may be a part reason for the downregulation of PTGIS.

**Fig. 8:**
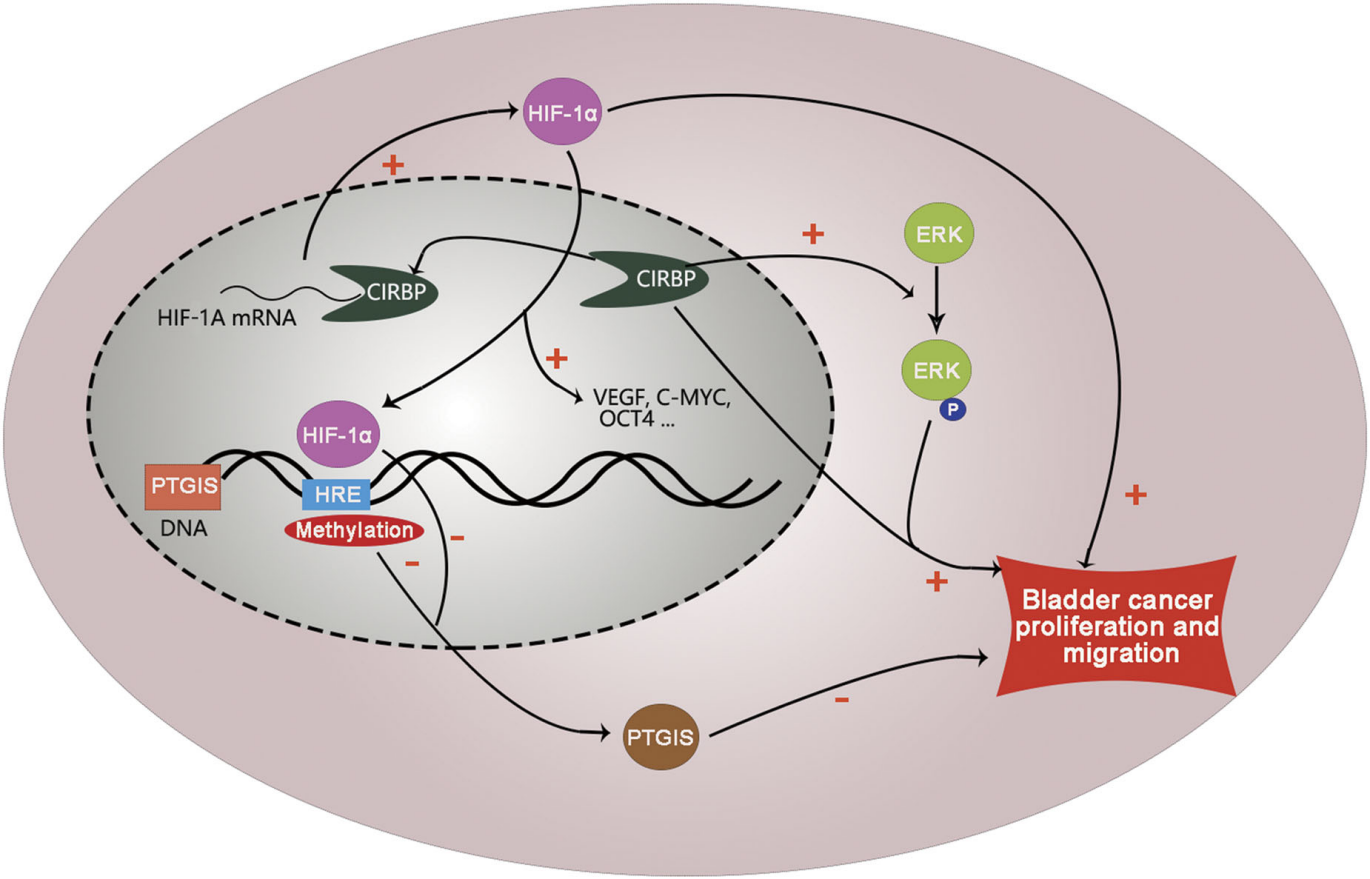
Mechanism diagram. Our study demonstrated that CIRBP could promote BCa cells proliferation and migration both in vitro and in vivo. Furthermore, CIRBP could increase HIF-1α expression by binding to the 3′-UTR of *HIF-1A* mRNA transcripts and increasing its mRNA stability in BCa cell line. HIF-1α could activate transcription of many oncogenes, such as VEGF, C-MYC, and OCT4. Our results showed that PTGIS is a HIF-1α target gene, which could suppress BCa cells proliferation and migration, and overexpression of HIF-1A could suppress the expression of PTGIS in BCa cells by binding to HRE sequence (5′-ccACGTGc-3′) of the PTGIS promoter region. Moreover, methylation of PTGIS gene promoter may be an important reason why PTGIS expression is downregulated by HIF-1α. And DNA methylation is also an important reason for obvious downregulation of PTGIS in BCa

Taken together, as shown in mechanism diagram (Fig. 8), our study demonstrated that CIRBP acts as an oncogene during BCa progression, which could promote BCa cells proliferation and migration both in vitro and in vivo. Furthermore, CIRBP could increase HIF-1α expression by binding to the 3′-UTR of HIF-1A mRNA transcripts and increasing its mRNA stability in BCa cell line. In addition, PTGIS is a HIF-1α target gene, which could suppress BCa cells proliferation and migration, and overexpression of HIF-1A could suppress the expression of PTGIS in BCa cells by binding to methylated HRE sequence (5′-ccACGTGc-3′) of the PTGIS promoter region. what’s more, further result suggested that knockdown of CIRBP could promote the expression of PTGIS, meanwhile knockdown of PTGIS could partially rescue siCIRBP induced inhibition of migration and proliferation in BCa cells

## Electronic supplementary material

Supplementary Information

supplementary Figures
