# Supplementary material for: CIRBP is a novel oncogene in human bladder cancer inducing expression of HIF-1α
Source: Cell Death Dis. 2018 Oct 12;9(10):1046. doi: 10.1038/s41419-018-1109-5 (PMC6185914; doi:10.1038/s41419-018-1109-5)
Supplement: Supplementary file 1 — Supplementary Information [file 41419_2018_1109_MOESM1_ESM.docx]

**CIRBP is a novel oncogene in human bladder cancer inducing expression of HIF-1α**

Mengxin Lu, Qiangqiang Ge, Gang Wang, Yongwen Luo, Xiaolong Wang, Wei Jiang, Xuefeng Liu, Chin-Lee Wu, Yu Xiao, Xinghuan Wang

**Supplementary Information**

**Supplementary Table S1. List of primers for qRT-PCR.**

| **Gene name** | **Forward primer (5’-3’)** | **Reverse primer (5’-3’)** | **Length (bp)** |
| --- | --- | --- | --- |
| CIRBP | AGGGCTGAGTTTTGACACCAA | ACAAACCCAAATCCCCGAGAT | 123 |
| GAPDH | TGCACCACCAACTGCTTAG | GATGCAGGGATGATGTTC | 176 |
| HIF-1A | GAACGTCGAAAAGAAAAGTCTCG | CCTTATCAAGATGCGAACTCACA | 124 |
| PTGIS | CTGTTGGGCGATGCTACAGAA | GCCTCAATTCCGTAAAGAGTCA | 113 |

**Supplementary Table S2. List of antibodies.**

| **Antigens** | **Species antibodies raised in** | **Dilution (WB)** | **Supplier** |
| --- | --- | --- | --- |
| CIRBP | Rabbit, polyclonal | 1:1,000 | Proteintech Group, USA, Cat. # 10209-2-AP |
| GAPDH | Mouse, monoclonal | 1:2,000 | Santa Cruz Biotechnology Inc., USA, Cat. #sc-365062 |
| p44/42 MAPK (Erk1/2) | Rabbit, monoclonal | 1:1,000 | Cell Signaling Technology, USA, Cat. # 4695 |
| Phospho-p44/42 MAPK (Erk1/2) (Thr202/Tyr204) | Rabbit, monoclonal | 1:1,000 | Cell Signaling Technology, USA, Cat. # 4370 |
| p38 MAPK | Rabbit, monoclonal | 1:1,000 | Cell Signaling Technology, USA, Cat. #8690 |
| Phospho-p38 (Thr180/ Tyr182) | Rabbit, monoclonal | 1:1,000 | Cell Signaling Technology, USA, Cat. # 4511 |
| E-cadherin | Rabbit, monoclonal | 1:500 | Cell Signaling Technology, USA, Cat. #3195 |
| N-cadherin, human | Rabbit, monoclonal | 1:500 | Cell Signaling Technology, USA, Cat. #13116 |
| Vimentin | Rabbit, monoclonal | 1:1,000 | Cell Signaling Technology, USA, Cat. #5741 |
| β-Catenin | Rabbit, monoclonal | 1:1,000 | Cell Signaling Technology, USA, Cat. #8480 |
| snail | Rabbit, monoclonal | 1:500 | Cell Signaling Technology, USA, Cat. # 3879 |
| HIF-1α | Mouse, monoclonal | 1:500 | Novus Biologicals, USA, Cat. # NB100-105 |
| PTGIS | Rabbit, polyclonal | 1:250 | Abcam, UK, Cat. #ab23668 |
| PPARγ | Rabbit, polyclonal | 1:500 | Abcam, UK, Cat. # ab45036 |
| Flag tag | Mouse, monoclonal | 1:1,000 | Proteintech Group, USA, Cat. # 66008-2-Ig |
| Anti-Mouse-IgG (H+L)-HRP | Goat | 1:10,000 | Sungene Biotech, China, Cat. #LK2003 |
| Anti-Rabbit-IgG (H+L)-HRP | Goat | 1:10,000 | Sungene Biotech, China, Cat. #LK2001 |

**Supplementary Table S3. List of primers for PTGIS promoter.**

| **Primer number** | **Forward primer (5’-3’)** | **Reverse primer (5’-3’)** | **Length (bp)** |
| --- | --- | --- | --- |
| Primer 1 | GGACCTCAGGGGAAGGTAGA | TGATCATGCCCTGGAACCAC | 126 |
| Primer 2 | CGAAAGCAAGGCAGGGTTTG | GGTTTCAGGTGAGGACCGAG | 149 |
| Primer 3 | TCCCACCTTGCACCTTCTTG | AGTCCCGCTCCAGTATCCC | 117 |
| Primer 4 | CGCGGCCCAAGCCAT | TCCGACCCCTGCGGAC | 187 |
| Primer 5 | GGAGAGGACTTTTGGGGAAG | CACTCCTCCTCCATGTTCGT | 143 |
| Primer 6 | CTGGGGACCTGGAGTTTTCC | AGGTGAGGATGAGGGGACTC | 136 |
| Primer 7 | GTCATCGCTACCTGGTGCTT | GACCCCCAGTTCTCATCAGC | 90 |

**Supplementary Figure S1**


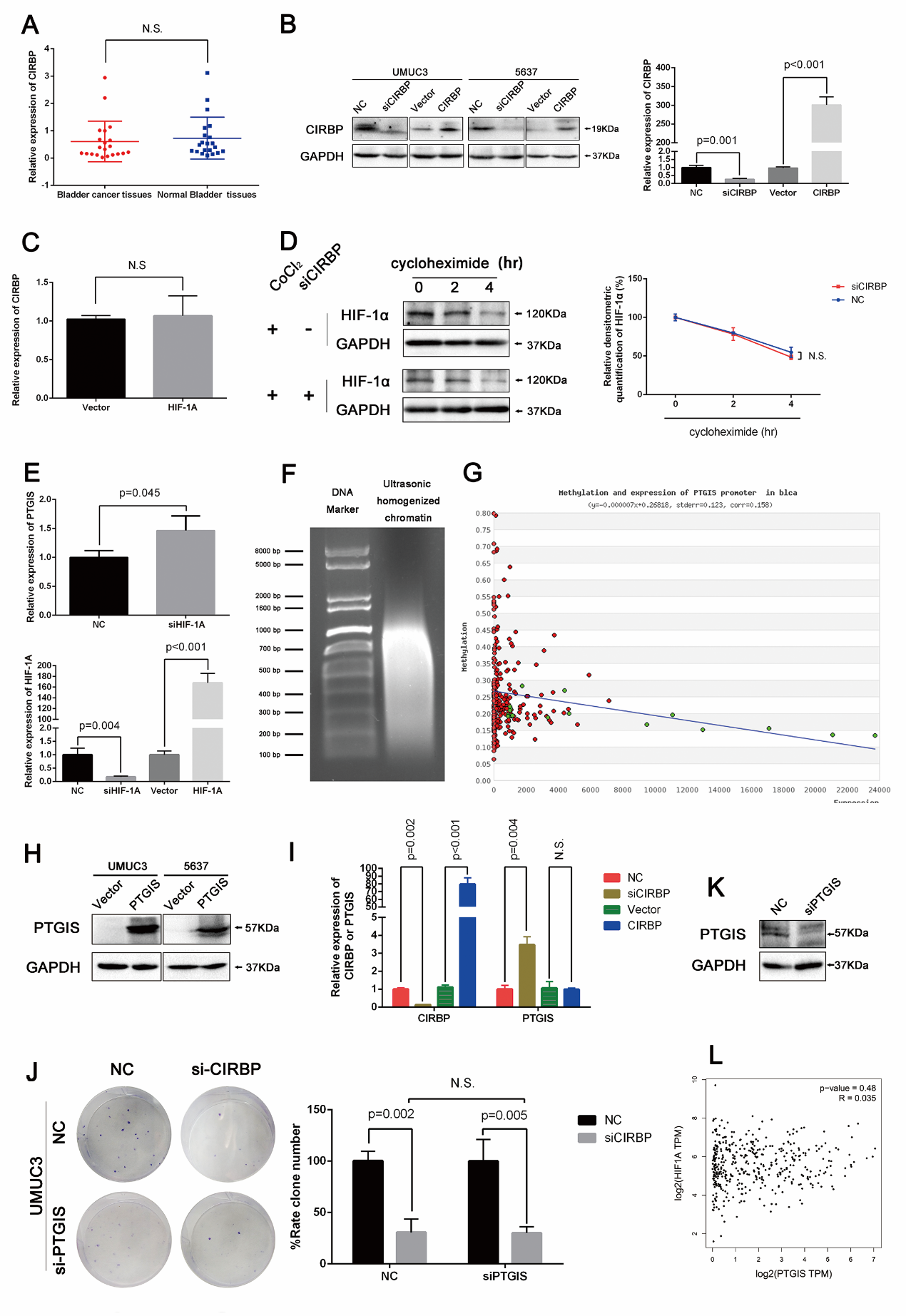


**Supplementary Figure S1. Relative gene expression and results from databases. (A)** qRT-PCR analysis exhibited the expression of *CIRBP* at the transcription level in BCa tissues compared with paracancerous tissues (n = 21). **(B)** Western blot and qRT-PCR analyses of CIRBP overexpression and knockdown efficiencies. **(C)** qRT-PCR analysis for the effects on the *CIRBP* mRNA levels of *HIF-1A* overexpression. **(D)** Cycloheximide assay to examine the effects of CIRPR on HIF-1α protein stability. **(E)** qRT-PCR analysis for the effects on the *PTGIS* mRNA levels of *HIF-1A* knockdown, and qRT-PCR analysis veriﬁcation of the *HIF-1A* overexpression and knockdown efficiencies. **(F)** DNA fragment size determined by electrophoresis on a 1% agarose gel. **(G)** MethHc database showed the negative correlation between PTGIS methylation levels and *PTGIS* mRNA expression levels in BCa. **(H)** PTGIS overexpression efficiencies verified by Western blot analysis. **(I)** qRT-PCR analysis for the effects on the *CIRBP* and *PTGIS* mRNA levels of CIRBP knockdown and CIRBP overexpression. **(J)** Rescue experiment of *siPTGIS*: clonogenic survival assay. **(K)** Western blot veriﬁcation of the *PTGIS* knockdown efficiency. **(L­)** GEPIA database showed no correlation between *HIF-1A* and *PTGIS* expression.
