# Supplementary figures and images for: CIRBP is a novel oncogene in human bladder cancer inducing expression of HIF-1α
Source: Cell Death Dis. 2018 Oct 12;9(10):1046. doi: 10.1038/s41419-018-1109-5 (PMC6185914; doi:10.1038/s41419-018-1109-5)

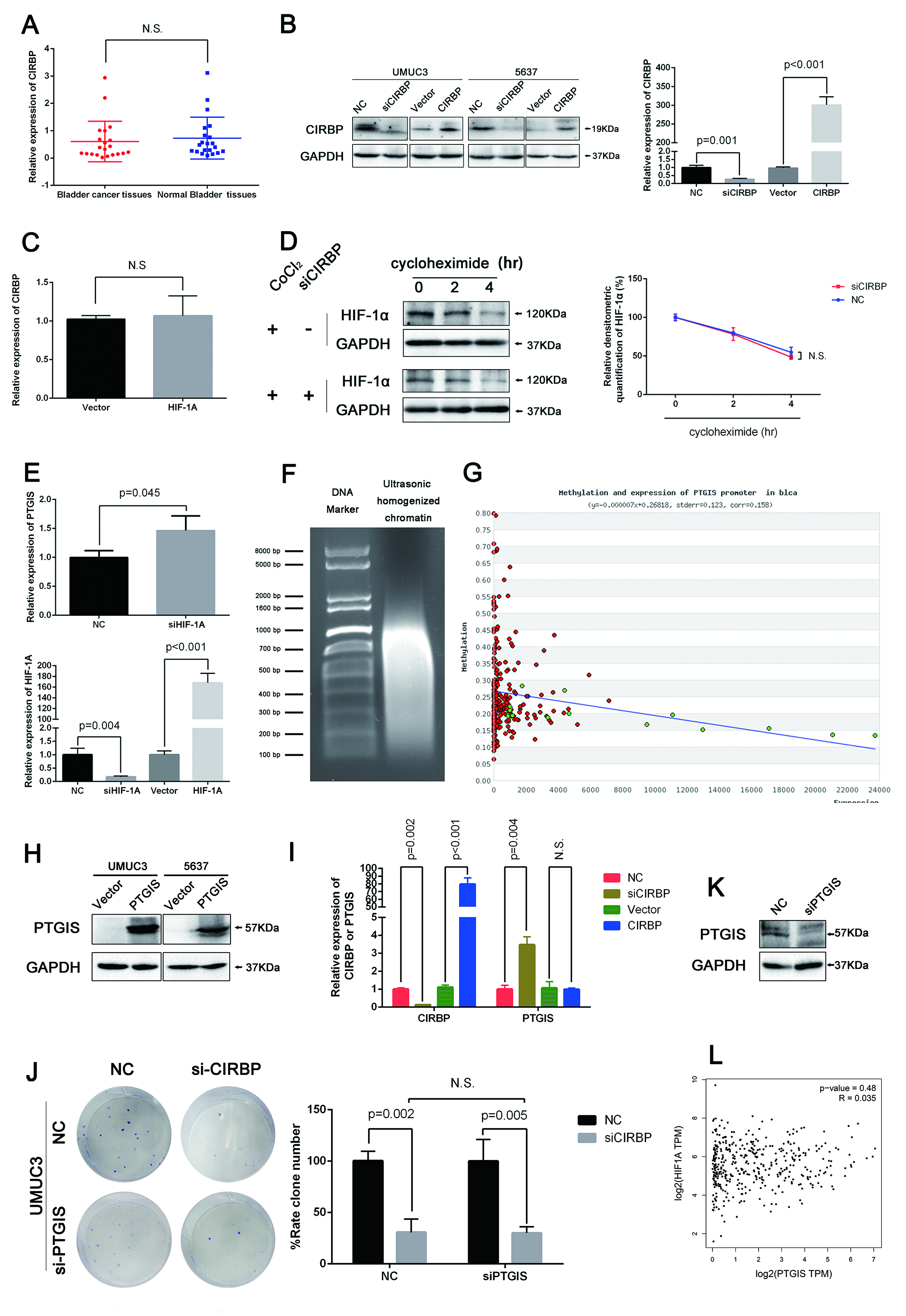

Supplement: Supplementary file 2 — supplementary Figures [file 41419_2018_1109_MOESM2_ESM.tif]
